# P-359. Healthcare Practitioners' Infection Prevention Strategies and Associated Factors in Primary Healthcare Facilities of Rohingya Refugee Camps in Bangladesh: A Qualitative Study

**DOI:** 10.1093/ofid/ofae631.560

**Published:** 2025-01-29

**Authors:** S M Murshid Hasan

**Affiliations:** Mahidol University, Dhaka, Dhaka, Bangladesh

## Abstract

**Background:**

Healthcare-associated infections (HAIs) pose significant challenges to both patients and healthcare providers. Rohingya refugee camps in Bangladesh are the most densely populated in the world. In the primary healthcare facilities of Rohingya refugee camps, healthcare-associated infections pose major barriers to safe and high-quality healthcare delivery among Rohingya refugees. The study's goal was to evaluate infection prevention strategies and associated factors among primary healthcare practitioners in Rohingya refugee camps.

**Methods:**

In 2023, we conducted a qualitative study among primary healthcare practitioners identified from 15 primary healthcare facilities in 15 Rohingya refugee camps. We conducted 60 in-depth interviews and six focus group discussions with community healthcare providers. We reviewed the collected data, developed a coding system, analyzed the data, and summarized findings according to the study objectives and themes.

**Results:**

Respondents were 60 % men and 40 % women, with a median age of 30 years, and 85% had higher secondary education. All respondents were employed for a median of 3 months (range,1-36 months). Only 42.5% of primary healthcare practitioners were knowledgeable about infection prevention strategies, and 37.2% practiced good infection prevention. Primary healthcare providers who had higher education and formal training in IPC were significantly more likely to have good infection prevention practices. Effective infection prevention practices were linked to the availability and accessibility of hand washing facilities and soap, proper use of personal protective equipment, appropriate cleaning and disinfection protocols, adherence to infection prevention guidelines, surveillance of healthcare-associated infections, and adequate knowledge of infection prevention measures of primary healthcare providers.

**Conclusion:**

Effective infection prevention and control practices are crucial for minimizing the risk of HAIs. This study assessed the infection prevention knowledge and practices, and their associated factors among primary healthcare practitioners in Rohingya refugee camps which would help develop and implement effective interventions to reduce HAIs within Rohingya refugee camps.

**Disclosures:**

**All Authors**: No reported disclosures

